# Early diagnostic and prognostic value of FEV₃/FEV₆ for COPD in underserved areas

**DOI:** 10.3389/fmed.2025.1627615

**Published:** 2025-08-08

**Authors:** Fei Li, Yancong Han, Xiaoling Yao, Fang Zhao, Qing Zhao, Weilin Liu

**Affiliations:** ^1^Health Care Department, The First Hospital of Hebei Medical University, Shijiazhuang, China; ^2^Health Care Department, The Eighth People’s Hospital of Hebei, Shijiazhuang, China; ^3^Respiratory Nephrology Department, The Eighth People’s Hospital of Hebei, Shijiazhuang, China; ^4^Science and Education Section, The Eighth People’s Hospital of Hebei, Shijiazhuang, China; ^5^Infectious Diseases Department, The Fourth Hospital of Hebei Medical University, Shijiazhuang, China

**Keywords:** FEV3, FEV6, COPD, early diagnosis, prognosis

## Abstract

**Background:**

Chronic obstructive pulmonary disease (COPD) presents a major global health burden. While FEV1/FVC is the diagnostic gold standard, its application is limited by patient compliance and procedural complexity. FEV3/FEV6 has been proposed as a simpler alternative for early COPD screening, but its diagnostic and prognostic value remains unclear. This study aims to evaluate the effectiveness of FEV3/FEV6 in diagnosing mild to moderate COPD and predicting patient outcomes.

**Methods:**

A total of 200 patients suspected of having mild to moderate COPD underwent FEV3/FEV6 and FEV1/FVC testing from June 2019 to June 2021 and were followed for 3 years. Correlation analysis and ROC curve analysis were conducted.

**Results:**

FEV6 correlated strongly with FVC (*r* = 0.981) and FEV3/FEV6 with FEV1/FVC (*r* = 0.928). FEV3/FEV6 showed high diagnostic accuracy (AUC = 0.953; sensitivity 87.92%; specificity 84.77%) and prognostic relevance (AUC = 0.783; sensitivity 80.3%; specificity 66.2%).

**Conclusion:**

FEV3/FEV6 is a reliable tool for early diagnosis and prognosis assessment in mild to moderate COPD.

## 1 Introduction

Chronic obstructive pulmonary disease (COPD) is a is typical respiratory ailment that is distinguished by long-lasting blockage of the airways and reduced lung function. COPD is listed as the third most typical reason for mortality worldwide, as stated by the World Health Organization (WHO) (1). In those 40 years of age and older, the prevalence of COPD is 10.1%, leading to approximately 3.23 million deaths annually. It’s noteworthy that 80% of these deaths occur in low- and middle-income countries, greatly increasing the burden of sickness and mortality worldwide (2). The primary risk factors for COPD include smoking, air pollution, and genetic predispositions (3, 4). Despite advancements in understanding the pathogenesis of COPD and improvements in clinical diagnosis and treatment strategies, the lack of prominent early symptoms often leads to delayed diagnosis, with many patients only being identified at more advanced stages of the disease (5). Consequently, finding workable solutions for early screening and diagnosis is crucial for enhancing the management and treatment outcomes of COPD patients.

While FEV1/FVC remains the gold standard for COPD diagnosis, its application faces several practical challenges, particularly in resource-limited settings. The test requires patients to exhale forcefully for a prolonged period (often >6 s) to achieve a complete FVC, which can be particularly challenging for elderly patients and those with severe respiratory conditions. This requirement often leads to poor patient compliance and potentially inaccurate results. Additionally, the sophisticated equipment and technical expertise required for accurate FVC measurement may not be readily available in primary healthcare settings or underserved areas.

In contrast, FEV3/FEV6 measurement presents clear clinical advantages. The shorter exhalation time of 6 s, compared to the extended period required for complete FVC, substantially improves patient cooperation and reduces exertion, particularly among elderly patients and those with impaired lung function. The standardized 6-s duration also streamlines the testing procedure, enabling broader implementation across primary healthcare facilities with varying resource levels. Recent research indicates that FEV3/FEV6 demonstrates heightened sensitivity in detecting early-stage COPD when compared to FEV1/FVC, especially in cases of subtle airway obstruction. This enhanced detection capability proves particularly valuable in identifying mild to moderate COPD cases that typically remain undiagnosed until symptoms progress to more severe stages.

Furthermore, the economic implications of implementing FEV3/FEV6 testing are significant. The simpler procedure requires less sophisticated equipment and minimal technical training, potentially reducing healthcare costs while maintaining diagnostic accuracy. This cost-effectiveness is particularly relevant in low- and middle-income countries, where COPD burden is highest but healthcare resources are often limited. The combination of improved patient compliance, simplified testing procedures, and potential economic benefits makes FEV3/FEV6 an attractive alternative for large-scale COPD screening programs, particularly in resource-constrained settings.

The FEV3/FEV6 ratio has attracted attention in clinical research as a new indicator in lung function testing. In lung function testing, measuring forced expiratory volume (FEV) is crucial for evaluating the extent of airflow obstruction. The FEV1/FVC ratio is a well-accepted and accurate way to diagnose COPD. It calculates the volume of air forcibly expelled in 1 sec relative to the total volume of air breathed (6). Still, the application of FEV1/FVC can be challenging in primary healthcare settings or underserved areas due to its complexity. In contrast, FEV3/FEV6, a newer lung function parameter, offers advantages such as simplicity and ease of use. Recent research has focused on its sensitivity and predictive value, highlighting its potential as an effective alternative (7, 8).

Early diagnosis is critical for the effective management of COPD. Accurate early detection can not only help slow disease progression and reduce mortality risk but also significantly enhance quality of life and lower treatment costs. Despite this, early diagnosis of COPD remains challenging. Although the traditional FEV1/FVC ratio is considered the gold standard, its lower detection rate in mild to moderate cases highlights the need for more effective screening and diagnostic methods. The emerging FEV3/FEV6 ratio, with its promising sensitivity and specificity for early COPD diagnosis, warrants further investigation (8–10). Additionally, managing and prognosticating COPD is complex, requiring a thorough assessment of disease severity, complications, and other factors. Currently, there is a lack of straightforward and accurate prognostic indicators. This study proposes FEV3/FEV6 as a potential prognostic factor, aiming to offer a new perspective for the long-term management of COPD.

The clinical value of the FEV₃/FEV₆ ratio stems from its sensitivity to early changes in pulmonary mechanics, particularly those affecting the small conducting airways. In the initial stages of COPD, airway narrowing and inflammation often begin in the peripheral small airways, which are less detectable by conventional FEV₁/FVC measurements. FEV₃ represents expiratory flow up to the third second, encompassing both large and small airway dynamics, while FEV₆ approximates forced vital capacity but requires a shorter, standardized effort. The FEV₃/FEV₆ ratio is thus capable of detecting delayed or incomplete expiratory flow that is characteristic of early airflow limitation and dynamic hyperinflation (11).

Additionally, several studies suggest that a declining FEV₃/FEV₆ ratio correlates with air trapping, increased residual volume, and reduced lung elastic recoil, all of which are hallmarks of progressive COPD (12). These pathophysiological changes have been linked to worsened exercise tolerance, higher exacerbation risk, and poorer long-term outcomes. Therefore, the FEV₃/FEV₆ ratio may not only offer greater sensitivity in the early diagnosis of COPD but also serve as a predictive marker for disease progression, facilitating timely intervention and optimized disease management strategies (13).

This study’s objective is to evaluate the practicality of FEV3/FEV6 in the early diagnosis of mild to moderate COPD and its potential for prognostic assessment. The goal is to enhance our understanding of early screening and prognostic tools for COPD and to provide more accurate diagnostic and treatment strategies for clinical practice.

## 2 Study design and methods

### 2.1 Study design

The research conducted a comparison between the sensitivity and specificity of FEV3/FEV6 and FEV1/FVC in order to diagnose mild to moderate COPD at an early stage. Additionally, the study examined the respective values of these measurements in assessing prognosis. The whole research process took place in compliance with the Declaration of Helsinki’s guiding principles and applicable ethical standards. Every participant willingly gave their informed permission, and the Ethics Committee of the Eighth People’s Hospital of Hebei gave its approval to the research plan [Approval No. (2022) KELUNSHEN No.1].

### 2.2 Study subjects

This study included 200 patients suspected of having mild to moderate COPD, who underwent pulmonary function tests at our hospital from June 2019 to June 2021. The patients, aged 35 to 75 years, were all referred from the respiratory outpatient clinic. While the main focus of this study was to assess the diagnostic and prognostic value of FEV₃/FEV₆ in mild to moderate COPD, a subset of patients with severe COPD was also included to provide additional context. The results for severe COPD patients will be presented as a supplementary analysis, with the primary emphasis on mild to moderate cases. Inclusion criteria were: (1) presence of typical COPD symptoms such as chronic cough, sputum, or dyspnea; (2) no history of other serious cardiopulmonary diseases; (3) no prior COPD-specific treatment. Exclusion criteria were: (1) acute respiratory tract infections or other acute disease exacerbations; (2) presence of severe cardiovascular disease, tuberculosis, or lung cancer; (3) cognitive impairment or inability to cooperate with pulmonary function tests.

### 2.3 Lung function tests

All participants underwent pulmonary function tests under standardized conditions. They were instructed to abstain from smoking avoid vigorous activity and wait at least 4 h before the test. A standard pulmonary function testing instrument (Spirolab III) was used and operated by trained professionals. Prior to testing, each participant inhaled 400 μg of salbutamol, a bronchodilator. Following a 15 min interval, forced expiratory volume in 3 sec (FEV3), forced expiratory volume in 6 sec (FEV6), forced expiratory volume in 1 sec (FEV1), and the forced vital capacity (FVC) were measured. The FEV3/FEV6 ratio was calculated using the measured values of FEV3 and FEV6. For conventional pulmonary function assessment, FEV1 and FVC were measured, and the FEV1/FVC ratio was calculated. A FEV1/FVC ratio fewer than 0.7 served as the COPD diagnostic standard (14). The GOLD classification of COPD (15) is detailed in Table 1.

**Table 1:** GOLD classification and study population breakdown.

Group	GOLD stage	% of predicted FEV₁	Characteristics	Number of patients (*n* = 200)	Percentage (%)
No COPD	-	≥0.7	No COPD	50	25%
Mild COPD	GOLD 1	≥80%	Mild airflow limitation	60	30%
Moderate COPD	GOLD 2	50–79%	Moderate airflow limitation	70	35%
Severe COPD	GOLD 3	30–49%	Severe airflow limitation	15	7.5%
Very Severe COPD	GOLD 4	<30%	Very severe airflow limitation	5	2.5%

The patients with mild, moderate, severe, and very severe COPD were classified based on their FEV₁/FVC ratio and corresponding GOLD stage. Patients with an FEV₁/FVC ratio ≥ 0.7 were classified as “No COPD”.

### 2.4 Diagnostic performance analysis

The receiver operating characteristic (ROC) curve analysis was utilized to evaluate the specificity and sensitivity of FEV3/FEV6 in the early diagnosis of mild to moderate COPD. For this analysis, only patients with confirmed mild to moderate COPD (GOLD 1–2) were considered positive cases, while patients not meeting the COPD diagnostic threshold (i.e., FEV₁/FVC ≥ 0.7) served as the control group. Patients with severe (GOLD 3) or very severe (GOLD 4) COPD were excluded from this ROC analysis to ensure alignment with the study’s diagnostic focus. The diagnostic efficacy was observed by calculating the receiver operating characteristic (AUC) curve. Hospitalization or COPD-related death was observed and recorded as a positive prognostic outcome.

### 2.5 Statistical analysis

The data analysis software used was SPSS 22.0. Mean ± standard deviation (SD) was the expression used to describe measurement data. The chi-square test accustomed to analyze the count data. To examine the relationship between FEV₃/FEV₆ and FEV₁/FVC, Pearson’s correlation coefficient analysis was performed. The prognosis and diagnostic utility of FEV3/FEV6 for mild to moderate COPD were assessed using ROC curve analysis. *p* < 0.05 indicates statistical significance.

## 3 Results

### 3.1 Basic characteristics of patients

The research included 200 patients with an average age of 58.4 ± 10.22 years, 134 of whom were males and 66 of whom were females, as seen in Table 2. Every patient matched the inclusion criteria and presented mild-to-moderate COPD symptoms, defined as chronic cough, sputum production, and dyspnea, in accordance with the Global Initiative for Chronic Obstructive Lung Disease (GOLD) classification. Specifically, mild COPD (GOLD 1) is characterized by a FEV₁ of 80% or more of predicted values, while moderate COPD (GOLD 2) is characterized by FEV₁ ranging from 50 to 79% of predicted values (16).

**Table 2:** Basic characteristics of the patients.

Basic characteristics	Values (*n* = 200)
Age (years)	58.4 ± 10.22
Gender (Male/Female)	134/66
Smoking history (%)	72%
Average smoking amount (pack-years)	25.3 ± 6.7
Chronic cough (%)	84%
Chronic sputum production (%)	76%
Dyspnea (%)	68%
FEV3 (L)	2.67 ± 0.78
FEV6 (L)	3.20 ± 0.81
FEV1 (L)	2.11 ± 0.64
FVC (L)	3.25 ± 0.83

### 3.2 Correlation analysis of FEV1/FVC and FEV3/FEV6

**Figure 1:**
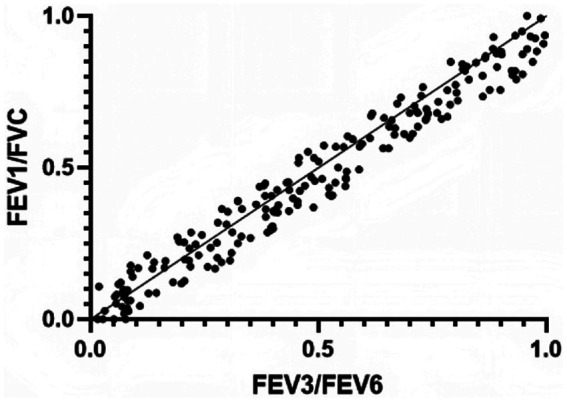
Scatter plot of FEV3/FEV6 VS FEV1/FVC with regression line.

Pearson’s correlation coefficient analysis revealed a strong positive correlation between FEV₁/FVC and FEV₃/FEV₆ (*r* = 0.928, *p* < 0.001). The scatter plot and regression analysis results are shown in Figure 1.

### 3.3 Diagnostic efficacy of FEV3/FEV6 in mild to moderate COPD

**Figure 2:**
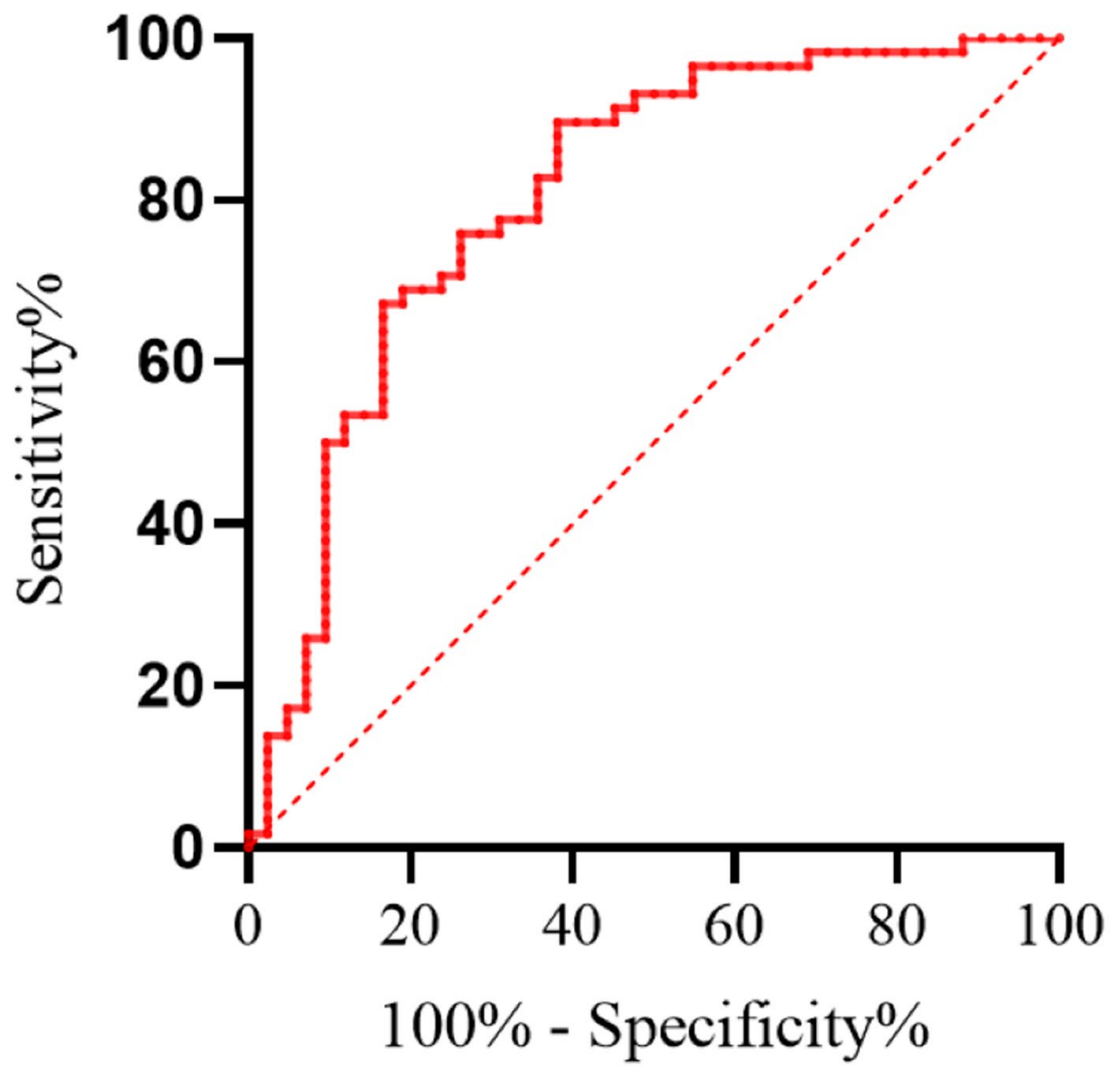
Receiver operating characteristic (ROC) curve for FEV₃/FEV₆ in diagnosing mild to moderate COPD. Only patients with GOLD stage 1–2 COPD and non-COPD individuals were included in this analysis; patients with GOLD stage 3–4 were excluded.

ROC curve analysis indicated that the area under the AUC for the diagnostic efficacy of FEV3/FEV6 in detecting mild to moderate COPD was 0.953 (95% CI: 0.927–0.979). The optimal tangent point for FEV3/FEV6 was at 0.735, where it achieved a specificity of 84.77%, sensitivity of 87.92%, and a Yoden index of 0.7269, as detailed in Table 3. Figure 2 displays the ROC curve for FEV3/FEV6.

**Table 3:** The clinical value of FEV3/FEV6 combined detection in the diagnosis of COPD.

Index	ROC AUC	95% CI	Sensitivity%	Specificity%	Yoden index
FEV3/FEV6	0.953	0.927–0.979	84.77	87.92	0.7269

### 3.4 Prognostic efficacy of FEV3/FEV6

**Figure 3:**
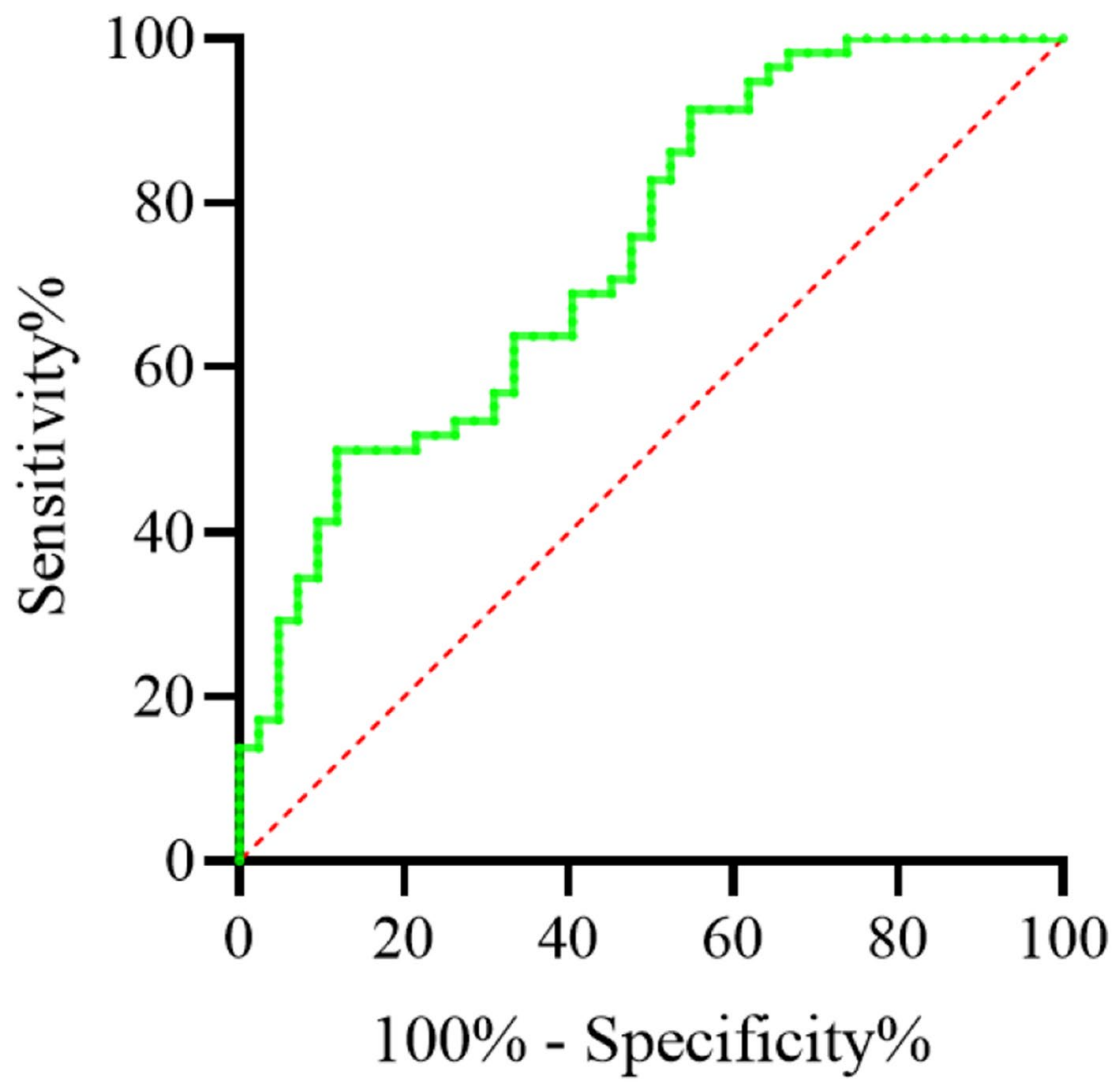
The ROC curve for FEV3/FEV6 in prognostic assessment.

During the 3-year follow-up period, 45 individuals were either hospitalized or died due to COPD-related complications. ROC curve analysis revealed that the AUC for the prognostic value of FEV3/FEV6 was 0.783 (95% CI: 0.713–0.853). The optimal cut-off point for FEV3/FEV6 was 0.710, with 66.2% specificity and 80.3% sensitivity, and a Youden index of 0.4652, as detailed in Table 4. Figure 3 illustrates the ROC curve for FEV3/FEV6 in prognostic assessment.

**Table 4:** Clinical value of FEV3/FEV6 combined detection in prognostic outcome.

Index	ROC AUC	95% CI	Sensitivity%	Specificity%	Youden index
FEV3/FEV6	0.783	0.713–0.853	80.3	66.2	0.4652

### 3.5 Performance of FEV3/FEV6 in different GOLD grades of COPD

**Figure 4:**
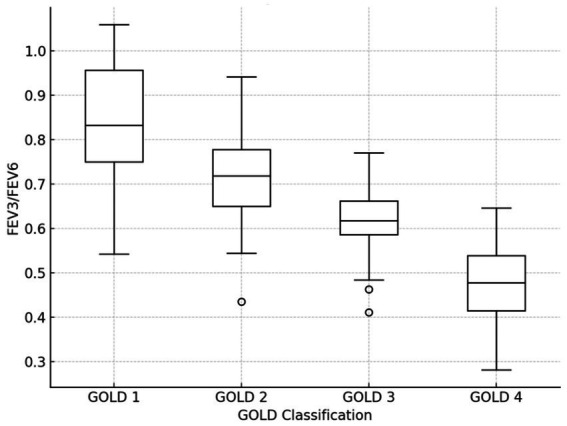
Boxplots of FEV3/FEV6 in different GOLD grades.

In this study, the main analysis focused on patients with mild to moderate COPD. However, we also present the performance of FEV₃/FEV₆ across the different GOLD stages (mild, moderate, severe, and very severe) to assess its broader utility in COPD diagnosis and prognosis. The results for severe and very severe COPD patients are discussed separately as part of the supplementary analysis to avoid confusion regarding the primary study aim. The distribution of FEV3/FEV6 in different COPD GOLD grades is seen in Table 5 and Figure 4. Figure 4 shows a boxplot of FEV3/FEV6 in different GOLD tiers.

**Table 5:** The distribution of FEV3/FEV6 in different COPD GOLD grades.

GOLD classification	FEV3/FEV6 (Mean± SD)
GOLD 1 (Mild)	0.85 ± 0.12
GOLD 2 (Moderate)	0.74 ± 0.10
GOLD 3 (Severe)	0.62 ± 0.09
GOLD 4 (Very Severe)	0.48 ± 0.07

## 4 Discussion

The main topic of this research is the application value of the FEV3/FEV6 ratio in the early diagnosis and prognosis of mild to moderate COPD. The results demonstrate that FEV3/FEV6 is highly correlated with the conventional lung function index FEV1/FVC. Additionally, FEV3/FEV6 exhibits high sensitivity and specificity for diagnosing mild to moderate COPD. Furthermore, this index also provides valuable insights for predicting the prognosis of COPD patients.

Early diagnosis and intervention are crucial for optimizing illness management and boosting the overall quality of life for patients with chronic diseases. This is especially important for chronic, progressive, and irreversible conditions like COPD. Traditionally, the FEV1/FVC ratio has been used as the gold standard for diagnosing COPD; however, this measure has its limitations (17). For instance, its sensitivity is relatively low in detecting mild to moderate COPD, which can result in missed opportunities for early diagnosis and timely intervention.

As an emerging indicator of lung function assessment, the FEV3/FEV6 ratio has received widespread attention in the last several years due to its simple and rapid operation. In this study, FEV3/FEV6 showed a high correlation with FEV1/FVC, which means that the FEV3/FEV6 ratio may serve as an effective early screening tool for COPD (18). In addition, the specificity and sensitivity of FEV3/FEV6 ratio indicate that it has certain clinical application value in mild to moderate COPD diagnosis. This finding is especially significant for primary hospitals and regions with limited resources, as the measurement of the FEV3/FEV6 ratio is more accessible and easier to implement compared to traditional methods (10). Regarding prognosis, while the sensitivity and specificity of FEV3/FEV6 may not match its diagnostic accuracy, it still offers valuable prognostic insights. This indicates that early lung function assessment using FEV3/FEV6 can aid not only in diagnosing COPD but also in providing partial predictions about disease progression and outcomes. Therefore, the FEV3/FEV6 ratio may be an effective technique for guiding the long-term management of COPD.

There are a few restrictions on this research, however. First, the findings’ capacity to be generalized could be influenced by the very little sample size. Second, the study did not account for all factors that could influence COPD diagnosis and prognosis, such as detailed lifestyle information, occupational exposure, and other environmental factors. Additionally, as the study was conducted at a single center, its findings need validation in a broader population. Future research should explore the use of the FEV3/FEV6 ratio in conjunction with other biomarkers or clinical indicators to enhance the accuracy of early COPD diagnosis and prognosis prediction. Despite these drawbacks, the research provides insightful information about the early diagnosis and management of COPD. The FEV3/FEV6 ratio, being a simple and user-friendly diagnostic tool, shows significant potential for early detection of mild to moderate COPD, improving diagnosis rates, and potentially enhancing patient prognosis through timely intervention. With further in-depth research, the FEV3/FEV6 ratio could become a practical and effective tool for COPD management.

Additionally, the influence of anterior chest wall conformation on spirometric indices such as FEV₆ and FEV₃/FEV₆ warrants further investigation. Previous studies have reported that individuals with anterior chest wall deformities may exhibit disproportionately reduced lung volumes and altered airflow parameters, even in the presence of mild to moderate obstructive disease (19). Although our study did not include an assessment of thoracic morphology, this anatomical factor may affect both the absolute values and interpretability of FEV₃/FEV₆, especially in early disease. Future studies should incorporate chest wall imaging or morphometric assessments to better delineate the relationship between chest conformation and simplified spirometric markers such as FEV₆ and FEV₃/FEV₆.

In summary, FEV3/FEV6 screening demonstrates significant potential for clinical application in the early diagnosis and prognosis assessment of mild to moderate COPD. Due to its simplicity and feasibility, the FEV3/FEV6 ratio should be more widely adopted in future COPD research and clinical practice. This broader use could lead to improved and more efficient medical care for COPD patients.

## Data Availability

The raw data supporting the conclusions of this article will be made available by the authors, without undue reservation.
